# Oxidative Stress Status in Post Stroke Patients: Sex Differences

**DOI:** 10.3390/healthcare10050869

**Published:** 2022-05-09

**Authors:** Mariacristina Siotto, Marco Germanotta, Massimo Santoro, Raffaella Canali, Simona Pascali, Sabina Insalaco, Valeria Cipollini, Dionysia Papadopoulou, Erika Antonacci, Irene Aprile

**Affiliations:** 1 IRCCS Fondazione Don Carlo Gnocchi ONLUS, 50143 Florence, Italy; mgermanotta@dongnocchi.it (M.G.); racanali@dongnocchi.it (R.C.); spascali@dongnocchi.it (S.P.); sinsalaco@dongnocchi.it (S.I.); vcipollini@dongnocchi.it (V.C.); dpapadopoulou@dongnocchi.it (D.P.); eantonacci@dongnocchi.it (E.A.); iaprile@dongnocchi.it (I.A.); 2 Energy and Sustainable Economic Development, Division of Health Protection Technologies ENEA-Italian National Agency for New Technologies, 00123 Rome, Italy; massimo.santoro@enea.it

**Keywords:** oxidative stress, antioxidant defense, Oxidative Status Index (OSI), hydroperoxides, stroke, rehabilitation

## Abstract

After a cerebral stroke insult, there is an overproduction of Reactive Oxygen Species (ROS), which overcome the antioxidant defenses, causing further tissues damage. The status of oxidative stress in stroke patients over time, particularly in those undergoing rehabilitation treatments, has been poorly investigated. We analyzed the oxidative stress status in 61 subacute stroke patients (33 females and 28 males) admitted to our rehabilitation center by measuring, in serum: hydroperoxides levels (d-ROMs), antioxidant activity (BAP test), and the relative antioxidant capacity (OSI index). We also analyzed patients for glucose levels and lipid profile. In addition, we analyzed the correlation between oxidative stress status biomarkers and motor deficits, disability, and pain. Almost all patients showed high or very high levels of d-ROMs, while BAP levels were apparently in the reference range of normality. Females had lower BAP values (females: 2478 ± 379; males: 2765 ± 590; *p* = 0.034) and lower OSI index (females: 5.7 ± 1.9; males: 6.8 ± 1.9; *p* = 0.043). Moreover, in the male group, the correlation with motor impairment and disability showed a worsened motor performance when oxidative stress is higher. Female group, on the other hand, had an unexpected different trend of correlation, probably due to an unbalanced systemic oxidative stress. Further research is needed to see if sex differences in oxidative stress status in subacute stroke patients persist after rehabilitation.

## 1. Introduction

Stroke is the principal cause of disability [1,2] and the second major cause of death worldwide, with a high burden on patients, their families, and health-care systems [3]. Patients after stroke have a very heterogeneous clinical spectrum, with variable and often partial recovery of motor function after a rehabilitation treatment [4]. Indeed, from 30 to 60% of patients present functional deficits of the paretic arm after a rehabilitation program, resulting in impaired activities of daily living [5].

During an ischemic insult, the brain–blood flow interruption causes multiple inflammatory immune responses and a general oxidative stress, which can damage the brain cells due to a secondary free radicals’ formation and lipid peroxidation [6]. This means that the brain is especially exposed to oxidative stress. 

Oxidative stress is defined as the imbalance between oxidant and antioxidant species, in favor of oxidants [7]. The most abundant free radicals are reactive oxygen species (ROS) and reactive nitrogen species (RNS), which are very highly reactive molecules, due to their unpaired electron(s) in their external shell. The source of free radicals can be endogenous (nicotinamide adenine dinucleotide phosphate, myeloperoxidase, lypoxigenase, angiotensin II, imbalance in essential metal homeostasis) or exogenous (pollution, alcohol, tobacco, heavy or transition metals, drugs, cooking radiation, etc.) [8,9]. Our antioxidant system counteracts the free radical toxicity and consists of endogenous antioxidants (enzymes: SOD, catalase, glutathione peroxidase; and non-enzymatic molecules: bilirubin, vitamin E, beta carotene, albumin, and uric acid) and exogenous antioxidants (Vitamin C, Vitamin E, phenolic antioxidants, oil lecithin, selenium, and zinc). Thus, oxidative stress status can be defined as the individual equilibrium between pro- and antioxidants [10]. This equilibrium varies with the general health status, specific disease condition, aging, or physical activity. 

Very few studies examined oxidative stress status in survivors of stroke who are undergoing rehabilitation [11,12,13] respect to stroke patients in the acute phase [14,15,16]. Thus, the aims of this study were to examine (i) the oxidative stress status in subacute stroke patients admitted to our rehabilitation center and (ii) the relationship between the oxidative stress status and motor impairment, disability, and pain, dividing subjects by sex. 

## 2. Materials and Methods

### 2.1. Sample

In this study, 61 patients with first stroke (33 females and 28 males), with a mean age of 68 ± 15 years, admitted to our rehabilitation department between 2019 and 2020 were consecutively enrolled.

The inclusion criteria were as follows: (i) first ischemic or hemorrhagic stroke, documented by magnetic resonance imaging (MRI) or computed tomography (CT); (ii) age between 55 and 85 years; (iii) time latency (within 6 months from stroke).

The exclusion criteria were as follows: (i) a previous stroke; (ii) behavioral and cognitive disorders and/or reduced compliance interfering with active therapy.

The study design was approved by the Ethical Committee of Don Carlo Gnocchi Foundation, Milan, Italy on 13 March 2019 (FDG_6_13/3/19). Written informed consent was obtained from all patients after a detailed explanation of the study’s aims and rehabilitation protocols (clinical trials identifier: NCT04223180).

### 2.2. Biochemical Analyses

The blood samples of patients were collected in the early morning (7:30–9:00 a.m.) after an overnight fast to standardize the assessment of those biochemical variables that are affected by the circadian cycle and food intake. Sera samples were separated by centrifugation (3000 rpm, 10 min, and 4 °C). They were then divided into 0.5 mL aliquots and rapidly stored at −80 °C. Subjects’ samples and reference samples were thawed just before the assay. All the analyses of the serum were performed in duplicate.

The colorimetric determination of Hydro-peroxides content (ROOH, principally) was assessed by d-ROMs test (Diacron, Grosseto, Italy) on an integrated analytical photometer (Free Carpe Diem, Diacron, Grosseto, Italy). This test measures the photometric variation between the byproduct of hydroperoxides with iron (RO∙ and ROO∙, as for Fenton reaction) and a substituted aromatic amine (solubilized in a chromogenic mixture). The values are expressed in arbitrary units (UCARR), with 1 UCARR corresponding to 0.08 mg/100 mL of hydrogen peroxide [17]. Reference values are between 250–300 UCARR, while 301–320 UCARR is considered borderline range, 321–340 UCARR low level oxidative stress, 341–400 UCARR middle level of oxidative stress, 401–500 UCARR high level of oxidative stress, and >500 UCARR very high level of oxidative stress (Diacron, Grosseto, Italy).

In the blood, the defense against noxious attack of reactive species, such as free radicals, is guaranteed by the antioxidant barrier, which includes exogenous (ascorbate, tocopherols, carotenoids, bioflavonoids, etc.) or endogenous (proteins, bilirubin, uric acid, cholesterol, GSH, etc.) compounds. Each mentioned antioxidant compounds possessed its action power to oppose, depending on reduction-oxidation potential, to the oxidant action of ROS. Such power is associated to the ability of plasma barrier components to give reducing equivalent (electrons or hydrogen atoms) to reactive species, avoiding the abstraction of hydrogen atoms from biomolecules and the generation of dangerous radical chains. It allows to measure the chemically active antioxidant capacity (scavengers) of the plasma barrier. In particular, it includes antioxidants of both exogenous nature (ascorbic acid and tocopherols) and endogenous nature (uric acid, bilirubin and albumin) [18]. The state of global antioxidant defenses and/or the effectiveness of specific antioxidant treatments in plasma was measured by a BAP test (Diacron, Grosseto, Italy) measured on an integrated analytical photometer (Free Carpe Diem, Diacron, Grosseto, Italy). This test is based on the capacity of a colored solution of ferric ions (Fe^3+^, R2 reagent) complexed to a chromogen to decolor when the ferric ions Fe^3+^ are reduced to ferrous ions (Fe^2+^). This reduction is generated by an adequate reducing system, that is, antioxidant as is plasma. Reference values in µmol/L of antioxidants is >2200, 2200–2000 is a borderline status, 2000–1800 is considered slight deficiency status, 1800–1600 deficiency status, and 1600–1400 high deficiency status, while <1400 is a very high deficiency status condition (Diacron, Grosseto, Italy).

The antioxidative/oxidative stress ratio was also calculated using the ratio equation: BAP/d-ROMs. In accordance with literature, we named this ratio the OSI index, which is an index of potential antioxidant capacity [19]. The criterion value for the BAP/d-ROMs ratio was set at 7.3. Accordingly, a value lower than 7.3 was defined as an oxidized type and a higher or equal one as a reduced type. A higher BAP/d-ROMs was considered preferable. For an extensive and elucidative review on oxidative stress indexes for the diagnosis of health or disease in humans, see the work of Sanchez-Rodriguez [20].

The glucose levels and lipid profile included Glucose, Cholesterol, HDL Cholesterol and triglycerides analyses (Diacron, Grosseto, Italy). Glucose was measured by an oxidase/peroxidase system; reference value: 70–105 mg/dL [21]. Total cholesterol was measured by means of oxidation from a cholesteroxidase to cholest-4-en-3-one; normal values are <200 mg/dL, borderline values are 200–240 mg/dL, and high value are >240 mg/dL [21]. Triglycerides was measured by a peroxidase-coupled method; reference value 40–165 mg/dL [21]. Direct HDL Cholesterol was measured with a new method of elimination, in which after an oxidative reaction eliminating VLDL and LDL, the HDL portion was transformed into a quinone derivative read at 600 nm [22]. The reference values are differentiated by sex for this assay: for males, normal levels are >55 mg/dL (females: >656 mg/dL); for males, levels of 35–55 mg/dL indicate a moderate risk (females: 45–65 mg/dL); and for males, values of 35 mg/dL indicate a high risk (females: 45 mg/dL). Finally, we calculated the ratio total cholesterol and HDL cholesterol ([Cholesterol]/[HDL cholesterol]) (normal reference range: for male < 5, for female < 4.5). 

### 2.3. Motor, Disability, and Pain Assessment

Patients were evaluated at admission in our rehabilitation center by means of: (i) the modified Barthel Index (BI), an ordinal scale used to measure performance in activities of daily living (ADL), ranging from 0 to 100, with lower scores indicating increased disability [23]; (ii) the Fugl-Meyer Assessment for upper extremity (FMA-UE) [24] to evaluate motor function; (iii) the upper-extremity subscale of the Motricity index (MI) [25] to evaluate limb strength; (iv) the Deambulation Index (DI), adapted form (eight-point scale) of the physical therapy part of the Patient Evaluation Conference System. The eight-point scale ranges from 0 (not assessed) to 7 [26]; (v) the Numerical Rating Scale (NRS), a unidimensional measure of pain intensity to diagnose and quantify pain in adults, in which a respondent selects a number from 0 (no pain) to 10 (extreme pain) that best reflects the intensity of their pain [27].

### 2.4. Statistical Analysis

Data were not normally distributed, according to the Shapiro–Wilk test, and therefore, non-parametric analysis was performed.

The values of d-ROMs and BAP of the sample group were compared using *t*-test with the identical test values measured in an Italian sample of 322 healthy volunteers (190 males and 132 females) subjected to a “health check” obtained from information on health status, physical measurements, and blood test [28]. 

To examine the relationship between biochemical data and demographic and clinical data, the Spearman rho correlation coefficients or the Mann–Whitney U test were used, as appropriate. This analysis was carried out in the whole sample, and for men and women, separately. Similarly, differences between men and women in biochemical, demographic, and clinical data were investigated using the Mann–Whitney U test or the chi-squared test.

For all the statistical analysis, a *p* value lower than 0.05 was deemed significant. Statistical analysis was performed using SPSS (IBM SPSS Statistics for Windows, Version 25.0. Armonk, NY, USA).

## 3. Results

### 3.1. Participants and Baseline Characteristics

For the study, 61 patients were enrolled and evaluated at admission to our rehabilitation center. Table 1 reports, in the whole group, and for females and males separately, the demographic and clinical characteristics of the sample. 

Comparing the two groups, the only differences were in terms of affected side (females had a larger proportion of left hemiplegia, while in males right and left hemiplegia had similar percentages) and hypertension (with a higher percentage in males). 

### 3.2. Oxidative Stress Biochemical Analyses

Oxidative stress serum analyses for the whole group, and divided between females and males, are reported in Table 2. A significative difference in BAP and OSI values were found between females and males, with antioxidant defense and OSI index lower in females (*p* = 0.034 and *p* = 0.043, respectively). On the contrary, no differences were found in terms of d-ROMs (*p* = 0.176). 

**Figure 1:**
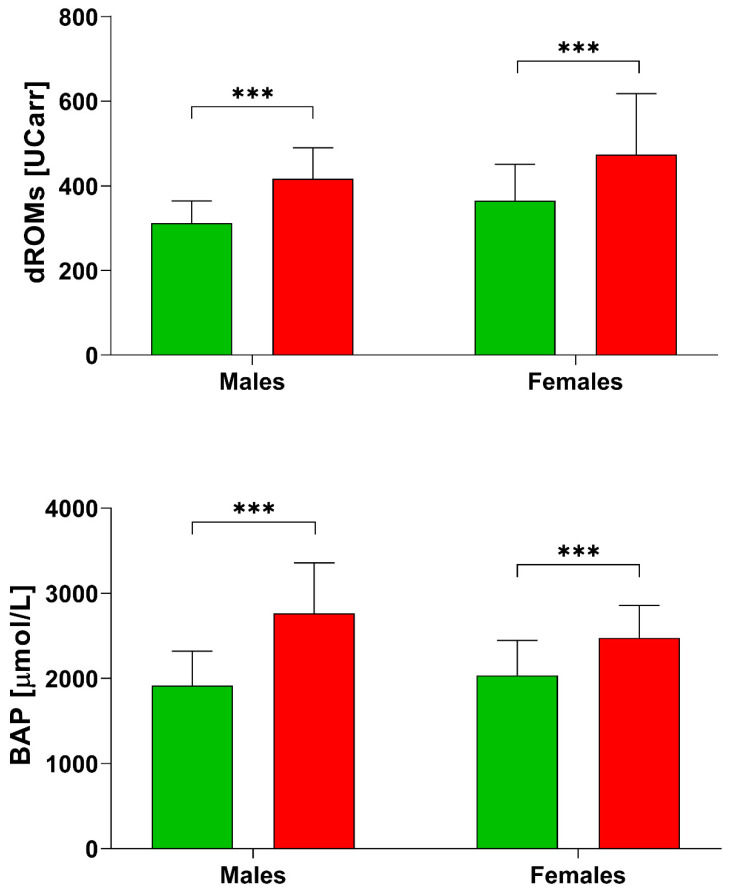
d-ROMs and BAP levels in healthy subjects [28] and post stroke patients divided by sex. *** *p* < 0.001 according to the *t*-test.

From the comparison with a group of Italian Healthy controls [28], we found that post stroke patients (Table 2) had higher values of d-ROMs (healthy females: 364.70 ± 85.90 UCARR; healthy males: 312.0 ± 52.30 UCARR; Figure 1). The BAP test resulted also higher in post stroke patients (healthy females: 2035.74 ± 412.28 µmol/L, while healthy males: 1945.03 ± 406.64 µmol/L), but our female group had lower BAP values compared to males (Table 2). 

**Figure 2:**
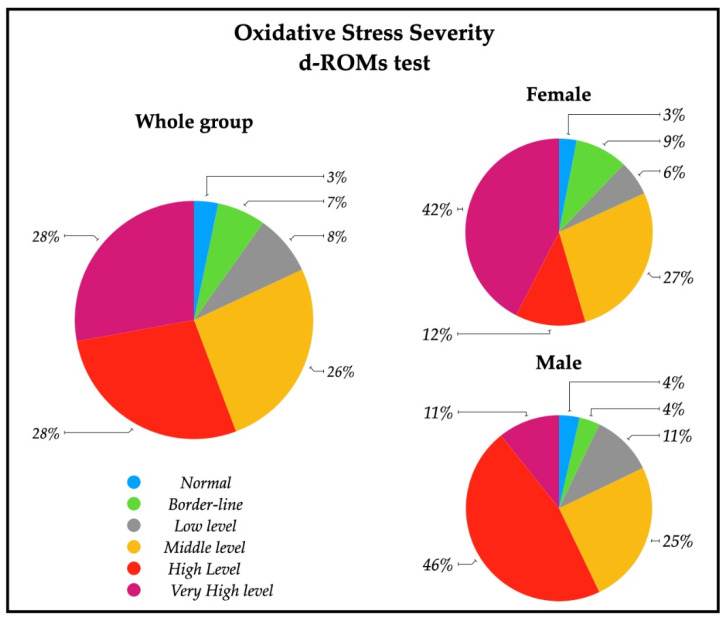
Pie charts representing the percentage of patients with oxidative stress severity in the whole group (*n* = 61), and in female patients (*n* = 33) and male patients (*n* = 28). Ranges are represented from normal to very high levels by means of d-ROMs test on serum. Normal (250–300 UCARR); Borderline (301–320 UCARR); Low level (321–340 UCARR); Middle level (341–400 UCARR); High level (401–500 UCARR); Very High level (>500 UCARR).

**Figure 3:**
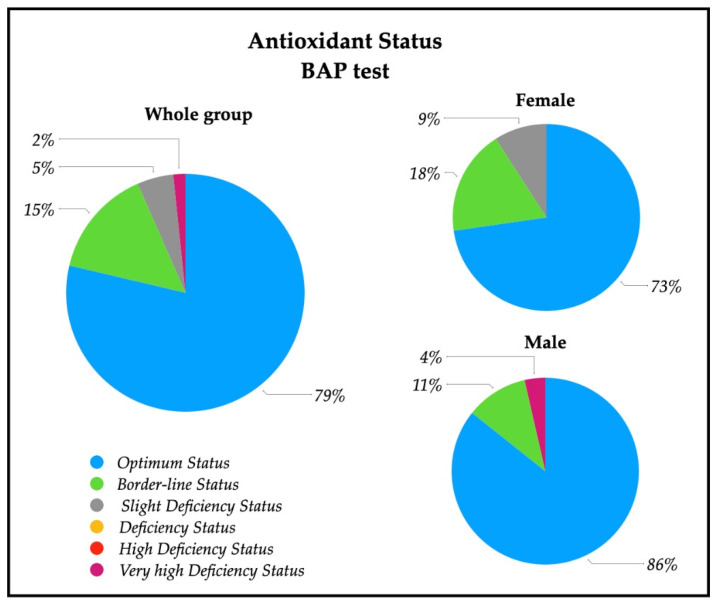
Pie charts of the percentage of patients with antioxidant status value in the whole group (*n* = 61), and in female patients (*n* = 33) and male patients (*n* = 28). Ranges are represented from optimum status to very high deficiency status measured by means of BAP test on serum. Optimum status (>2200 µmol/L); Borderline status (2200–2000 µmol/L); Slight deficiency status (2000–1800 µmol/L); Deficiency status (1800–1600 µmol/L); High Deficiency Status (1600–1400 µmol/L); Very high Deficiency Status (<1400 µmol/L).

A descriptive analysis of the oxidative stress severity ranges, measured by d-ROMs serum test, are reported in Figure 2; a significative percentage of patients had high (28%) and very high (28%) percentages of oxidative stress severity. In particular, 43% of females had a very high oxidative stress level, while a similar percentage of males (43%) showed a high oxidative stress. The descriptive analysis of the antioxidant status ranges analyzed with BAP serum test are depicted in Figure 3, and revealed that most of the patients (79%) had an optimum power of counteracting oxidative stress (86% of males and 73% of females). 

The OSI index values was below the cut-off of 7.3 in 74% of patients (76% of females and 71% of males), indicating an insufficient antioxidant power with respect to hydroperoxide circulation. 

### 3.3. Glucose Level and Lipid Profile Biochemical Analysis

Table 3 shows the biochemical analysis of the glucose levels and lipid profiles for the whole group, and divided by female and male. At the moment of admission in our structure, all of the patients had normal glucose, cholesterol, and triglyceride levels, with no sex differences. Only HDL cholesterol levels were different between males and females; while both were within normal reference ranges, males HDL cholesterol was lower and near the borderline value of 55 mg/dL, but the cholesterol ratio was normal.

### 3.4. Correlation between d-ROMs, BAP, and OSI with Glucose Levels and Lipid Profile, Days from Index Stroke to Enrollment, and Motor, Disability, and Pain Assessment

The analysis of correlations between the hydroperoxides levels measured by means of d-ROMs levels is reported in Table 4. d-ROMs showed a positive correlation with glucose in the whole group and in female group. Moreover, a negative correlation with days from index stroke to enrollment, in the whole sample and in males. No correlations were found with the glucose levels and lipid profile, nor with pain, as measured by the NRS scale. In the whole group, a positive correlation was found between d-ROMs values and MI and FMA-UE; in females, d-ROMs values correlated positively with DI, MI, and FMA-UE; in males, a negative correlation was found between d-ROMs and BI and DI.

The analysis of correlations between antioxidant capacity by means of BAP levels (Table 5) showed in the male group a negative correlation with triglycerides and a positive correlation with the NRS. Correlation with motor assessment: in the whole group, BAP was found to be negatively correlated with the FMA-UE, while in the female group, a negative correlation was found with BI and DI.

The OSI index correlated negatively with glucose and triglycerides in the whole group and in the male group. Moreover, in the whole group, a negative correlation was found with FMA-UE; in the female group, negative correlations were found with BI, DI, and MI, while positive correlations were found with BI and DI in the male group (Table 6).

No differences were found between smokers and no-smokers in oxidative stress markers. A negative correlation between d-ROMs and time from stroke onset was seen in the whole group and in the male group, showing that the further the distance from insult the lower the hydroperoxides levels.

## 4. Discussion

The analysis of oxidative stress status in the subacute stroke patients enrolled for this study revealed that systemic hydroperoxides levels were altered, as expected: 56% of the subjects had high and very high d-ROMs serum content (Figure 2, Table 2).

After an ischemia or a hemorrhagic brain injury, there is a massive production of ROS—as revealed by hydroperoxides in circulation—which can produce multiple reactions of radicals damaging the cells; lipid content of membrane cells is particularly susceptible to ROS attacks, because lipid peroxidation involves the inactivation of membrane enzyme and the destruction of the structural protein [29,30]. Several studies showed that acute and subacute ischemic stroke patients had increased levels of oxidative stress [11,12,31] and clinical severity of stroke was demonstrated to be correlated with increased serum hydroperoxide concentrations, measured with the d-ROMs test [16]. Moreover, free radicals also prevent recovery, which makes them an important post stroke therapeutic target [32]. 

Patients were admitted to our center after a certain time since stroke insult and we found negative correlation between d-ROMs and time from stroke onset in the whole group and in the male group, showing that the further the distance from insult the lower the hydroperoxides levels, as described in the literature [33]. Moreover, we found unexpectedly that the antioxidant levels were optimum (Table 2). In the whole group under study, in fact, the BAP test revealed a good antioxidant capacity in 79% of subjects (Figure 3). This result appears to point to a “counter-balanced oxidative stress” status, in which patients tend to neutralize the oxidative stress generated by stroke insult with an apparently good biological antioxidant capacity. 

To investigate the redox balance more thoroughly, we calculated the OSI index for each patient; this index showed that the relative antioxidant capacity was not so effective, since 71% of the whole group had OSI under the cut-off (Table 2). Thus, the oxidant insult it is probably not sufficiently opposed by endogenous antioxidant capacity of patients. This scenario, however, is potentially harmful, because the oxidative stress could worsen if ROS production is not promptly removed or reduced [29]. There was no difference between smokers and no-smokers in oxidative stress biomarkers, and smokers were a very small percentage of patients, so we can exclude that hydroperoxides increase is related to smoking. It is also worth noting that the high mean age of our sample (68 ± 15 years; Table 1) can be a cause of worse antioxidant capacity. 

We then compared our data to those of 322 Italian controls [28] and we found that our group had higher d-ROMs and BAP measurements (Figure 1). This “comparison group” was well defined in terms of age and gender, with no differences in d-ROMs values for age, and females having higher d-ROMs values. Antioxidant defenses decreased with age in the BAP test, with higher BAP values in females. The authors also divided females into premenopausal and postmenopausal groups but found no statistical differences [28]. 

Similarly, d-ROMs values were significantly different between 105 males and 185 females in another group of 290 “apparently healthy” Italians over 60, while PAT, which is an evolution of the BAP test, did not show significant differences [34]. 

In our patients, no differences were found in d-ROMs between males and females, but 42% of females had very high values of d-ROMs with respect to 11% of males (Figure 2). Females showed, instead, a lower level of BAP respect to males, with a lower percentage of normal levels of systemic antioxidants (Table 2, Figure 3). These findings show that oxidative stress status is different in the male and female group, but differently respect to healthy subjects. The higher systemic hydroperoxides levels measured with d-ROMs are most likely due to the stroke insult in both sexes, maintaining the same pattern of healthy subjects (with higher values in female), but our female group’s antioxidant defense reservoirs appear lower to be lower.

Stroke incidence has long been known to be higher in males than females around the world; this sexually dimorphic epidemiology persists well past menopause until it is overshadowed by the effects of age [35,36]. Males have a higher age-adjusted stroke incidence than females [37], but females have not seen the same reduction in stroke rates as males, according to recent research [38]. Women’s higher stroke rates may be due to longer life expectancies, but sex differences in stroke incidence rates may also play a role. A recent study on the X and Y chromosomes suggested that the second X chromosome has a negative effect that is only visible after reproductive senescence [39,40]. This suggests a complex interaction between aging, ischemia, and the sex chromosome, and that sex should be considered in the prevention and treatment of stroke [35,38,41].

The analysis of glucose levels and lipid profile, at the admission to our rehabilitation center, showed that all subjects had normal glucose, cholesterol, and triglyceride levels (Table 3), with no sex differences and no statistical differences between sex. Only HDL cholesterol levels differed between males and females being both within normal reference ranges; male HDL cholesterol was lower and near the borderline value of 55 mg/dL, but the total cholesterol/HDL cholesterol ratio remained within normal reference ranges. Brunelli et al. [42] analyzed sex differences in biomarkers in 195 Italian healthy volunteers, and found that males had lower HDL cholesterol values at the baseline, although the differences were not significant (males: 48.69 ± 14.10 mg/dL; female: 61.06 ± 11.78 mg/dL). In a more recent epidemiological study by Menotti [43], the analysis of 25,272 males (with median age of 51) and 21,895 females (median age 49) revealed higher levels in females.

The analysis of correlations with oxidative stress indices showed a positive correlation between d-ROMS and glucose in females and a negative correlation between BAP and triglycerides in males. These findings deserve further study to investigate better the relationship between glucose and lipid profile, as well as oxidative stress biomarkers, in subacute stroke male and female patients admitted to rehabilitation centers, considering that subjects after a stroke insult are often treated pharmacologically for hypercholesterolemia and diabetes. Note that among the comorbidities the hypertension is significantly more present in males than females and we do not able to exclude that this disease or the anti-hypertension drug assumption can have an influence on the oxidant stress status.

Another result of our study is that the analysis of correlation of d-ROMs and OSI index with motor assessment outcome showed very singular results: a substantial difference was observed between the female and male group. In the male group, the Barthel Index and Deambulation Index negatively correlated with d-ROMs and positively with the OSI index, showing that the higher the oxidative stress status was, the worse the motor function (Table 3 and Table 5). Females had a totally different trend: the higher the d-ROMs values were, the better the ability to walk (measured using the Deambulation index) and the better the upper limb muscle strength performance (measured using the Motricity Index and Fugl-Meyer Assessment); the lower the antioxidant capacity was, the better the ability in daily activities and the better the deambulation (higher score in Barthel index and Deambulation index); the lower the OSI index was, which accounts for the antioxidant capacity index, the higher all the motor assessment outcomes were, i.e., Barthel index, Deambulation index, Motricity index, and the Fugl-Meyer Assessment (Table 3, Table 4 and Table 5). Males seem less distressed, and the oxidative stress status showed a significant correlation with motor ability. This particular behavior of the oxidative stress status in female is not associated with a higher disability or worse performance with respect to males because we found no significant differences in the relative outcomes. In our opinion, it is possible that females, after a certain level of oxidative stress, are no longer able to balance it, having an inadequate antioxidant capacity, and this implies that there is no correlation with motor assessment outcomes. This hypothesis has to be confirmed by measuring oxidative stress before and after rehabilitation, in order to clarify this point. In fact, the rehabilitation programs have been proven to be crucial in diminishing oxidative stress, lowering oxidative stress biomarkers in plasma [11,13,44,45]. In particular, a study on 29 subacute-phase post stroke patients reported levels of d-ROMs and BAP in plasma immediately before and after the exercises at admission and after a rehabilitation program, comparing an exercise group with a control one [12]. D-ROMs levels were significantly decreased and BAP levels significantly increased at rest only in the exercise group. It is interesting to notice that the levels of d-ROMs, measured immediately after the 1-h exercise, were always higher than before exercise. It is well known that high-intensity physical exercise is associated with increased production of reactive oxygen species, able to consume endogenous antioxidants and eventually able to damage biological molecules and key cellular components. Therefore, the balance between beneficial and potentially harmful effects of exercise might be of particular importance in the elderly, in which nutritional deficiencies, sedentary lifestyle, and comorbidity commonly concur with a depletion of the antioxidant reservoir of the organism and increased susceptibility to oxidative stress [46]. On the other hand, at rest, levels tend to diminish, showing the very positive effect of antioxidant activity exerted by exercise training. In this context, rehabilitation should be considered crucial not only for functional improvement and functional maintenance after stroke, but also for improving antioxidant capacity and attenuating systemic oxidative stress. 

One limitation of this study is that we did not measure inflammation biomarkers or hormonal biomarker such as estrogen, which deserves future investigation for its correlation with oxidative stress. Another important limitation is that we tested only d-ROMs and BAP: the analysis of other biomarkers and an analysis on iron dysmetabolism need to be performed to extend and improve the study on oxidative status imbalance in post stroke patients. Furthermore, this study was carried out on a limited number of subjects and without an internal healthy control group; a study with a wider sample including a control group is planned to confirm these data. Moreover, we had no information about the cerebral area affected from the stroke, and this could be a critical point to evaluate the differences existing in oxidative stress among males and females. 

From these preliminary data it is clear that further research is necessary to investigate the role of rehabilitation on oxidative stress status in post stroke patients and to evaluate if sex is a distinguishing factor of the response of the antioxidant ability to treatment. It is important to underline that the clinical picture after stroke can be heterogeneous and its evolution as well as the response to rehabilitation treatments can be very different in individuals, and in males and females, despite similar clinical status at the onset [47,48,49]. Reducing oxidative stress generated by free radicals is an important issue to consider in order to limit subsequent stroke insults. Moreover, further research is necessary to investigate the potential role of a specific diet or of antioxidant supplementation to overcome the oxidative imbalance reported in this study.

## Figures and Tables

**Table 1:** Baseline characteristics of the whole group (*n* = 61) and of females (*n* = 33) or males (*n* = 28). Data are reported in mean ± standard deviation or number and percentage (%).

Baseline Characteristics	Whole Group (*n* = 61)	Females (*n* = 33)	Males (*n* = 28)	*p* Value
Age (years)	68 ± 15	72 ± 13	65 ± 17	0.176
*Index stroke type*				
Ischemic	48 (78.7%)	29 (87.9%)	19 (67.9%)	0.057
Hemorrhagic	13 (21.3%)	4 (12.1%)	9 (32.1%)	
*Affected side*				
Right	24 (39.3%)	9 (27.3%)	15 (53.6%)	0.036 *
Left	37 (60.7%)	24 (72.7%)	13 (46.4%)	
Smoking	9 (14.8%)	5 (15.1%)	4 (14.3%)	0.9243
*Comorbidities*				
Hypertension	42 (68.9%)	19 (57.6%)	23 (82.1%)	0.039 *
Type 2 Diabetes	16 (19.7%)	6 (18.2%)	6 (35.7%)	0.7506
Dyslipidemia	10 (16.4%)	6 (18.2%)	4 (14.3%)	0.6821
Heart disease	23 (37.7%)	15 (45.5%)	8 (28.6%)	0.1752
Time from stroke onset (days)	110 ± 37	110 ± 40	110 ± 33	0.745
Numerical Rating Scale (pain)	3 ± 3	4 ± 3	3 ± 2	0.093
*Motor Assessment*				
Modified Barthel Index (0–100)	47.0 ± 20.4	46.2 ± 20.4	48.0 ± 20.7	0.592
Deambulation Index	2.0 ± 1.7	1.8 ± 1.7	2.1 ± 1.8	0.400
Motricity Index	39.2 ± 26.6	39.8 ± 26.8	38.2 ± 27.1	0.881
Fugl-Meyer Assessment	20.3 ± 17.0	23.4 ± 17.4	15.5 ± 15.7	0.222

* *p* value < 0.05.

**Table 2:** Oxidative stress biochemical analyses.

Biochemical Analyses	Whole Group (*n* = 61)	Female (*n* = 33)	Male (*n* = 28)	*p* Value
d-ROMs (UCARR)	448 ± 119	474 ± 144	417 ± 73	0.176
BAP (µmol/L)	2610 ± 504	2478 ± 379	2765 ± 590	0.034 *
OSI = BAP/d-ROMs ratio	6.2 ± 2.0	5.7 ± 1.9	6.8 ± 1.9	0.043 *

Data are reported as mean ± standard deviation; * *p* value < 0.05.

**Table 3:** Glucose levels and lipid profile.

Biochemical Analyses	Whole Group (*n* = 61)	Female (*n* = 33)	Male (*n* = 28)	*p* Value
Glucose (mg/dL)	97.8 ± 42.2	95.1 ± 46.5	100.9 ± 37.2	0.473
Cholesterol (mg/dL)	119.0 ± 30.0	124.3 ± 28.3	113.0 ± 31.4	0.119
HDL Cholesterol (mg/dL)	64.4 ± 19.8	70.3 ± 19.7	57.5 ± 17.8	0.016 *
Cholesterol ratio (Cholesterol/HDL Cholesterol)	2.0 ± 0.7	1.9 ± 0.6	2.1 ± 0.7	0.300
Triglycerides (mg/dL)	113.7 ± 37.5	108.3 ± 36.4	119.9 ± 38.4	0.293

Data are reported as mean ± standard deviation; * *p* value < 0.05.

**Table 4:** Correlation between the systemic hydroperoxides, measured by d-ROMs, and the glucose levels and lipid profile, days from index stroke to enrollment, pain scale, and motor and disability assessment in the whole group, and in female and male separately.

	d-ROMs					
	Whole Group (*n* = 61)		Female (*n* = 33)		Male (*n* = 28)	
	Spearman Rho	*p* Value	Spearman Rho	*p* Value	Spearman Rho	*p* Value
Glucose (mg/dL)	0.403 *	0.002	0.486 *	0.006	0.312	0.113
Cholesterol (mg/dL)	0.193	0.146	0.275	0.134	−0.059	0.770
HDL Cholesterol (mg/dL)	−0.054	0.689	−0.266	0.148	0.168	0.403
Triglycerides (mg/dL)	0.157	0.238	0.174	0.350	0.304	0.124
Time from Stroke onset (days)	−0.305 *	0.017	−0.258	0.147	−0.439 *	0.019
Numerical Rating Scale (pain)	−0.055	0.705	−0.226	0.258	0.049	0.827
*Motor/disability Assessment*						
Barthel Index	−0.004	0.975	0.254	0.154	−0.485 *	0.009
Deambulation Index	0.112	0.393	0.493 **	0.004	−0.505 *	0.006
Motricity Index	0.351 *	0.021	0.458 *	0.019	−0.009	0.974
Fugl-Meyer Assessment	0.379 *	0.016	0.495 *	0.014	−0.054	0.843

* *p* value < 0.05; ** *p* value < 0.005.

**Table 5:** Correlation between the total antioxidant capacity, measured by BAP, and the glucose levels and lipid profile, days from index stroke to enrollment, pain scale, and cognitive and motor assessment in the whole group, in females, and in males.

	BAP					
	Whole Group (*n* = 61)		Females (*n* = 33)		Males (*n* = 28)	
	Spearman Rho	*p* Value	Spearman Rho	*p* Value	Spearman Rho	*p* Value
Glucose (mg/dL)	−0.040	0.763	0.137	0.463	−0.245	0.217
Cholesterol (mg/dL)	−0.012	0.930	−0.071	0.703	−0.115	0.570
HDL Cholesterol (mg/dL)	0.050	0.712	0.202	0.274	0.120	0.550
Triglycerides (mg/dL)	−0.247	0.062	0.073	0.695	−0.569 **	0.002
Time from stroke onset (days)	0.042	0.749	−0.088	0.627	0.167	0.395
Numerical Rating Scale (pain)	0.008	0.957	−0.066	0.745	0.436 *	0.043
*Motor Assessment*						
Barthel Index	−0.121	0.353	−0.416 *	0.016	0.076	0.700
Deambulation Index	−0.088	0.505	−0.391 *	0.027	0.125	0.527
Motricity Index	−0.111	0.480	−0.261	0.197	0.140	0.591
Fugl-Meyer Assessment	−0.317 *	0.046	−0.379	0.068	−0.252	0.347

* *p* value < 0.05; ** *p* value < 0.005.

**Table 6:** Correlation between the OSI index and glucose levels and lipid profile, days from index stroke to enrollment, pain scale, and cognitive and motor assessment in the whole group, in females, and in males.

	OSI Index					
	Whole Group (*n* = 61)		Females (*n* = 33)		Males (*n* = 28)	
	Spearman Rho	*p* Value	Spearman Rho	*p* Value	Spearman Rho	*p* Value
Glucose (mg/dL)	−0.277 *	0.035	−0.237	0.198	−0.415*	0.031
Cholesterol (mg/dL)	−0.082	0.538	−0.242	0.189	0.200	0.318
HDL Cholesterol (mg/dL)	0.078	0.560	0.306	0.094	0.001	0.997
Triglycerides (mg/dL)	−0.303 *	0.021	−0.153	0.410	−0.609 **	0.001
Time from Stroke onset (days)	0.211	0.102	0.140	0.436	0.295	0.128
Numerical Rating Scale (pain)	0.072	0.624	0.188	0.347	0.167	0.458
*Motor Assessment*						
Barthel Index	−0.015	0.912	−0.392 *	0.024	0.422 *	0.025
Deambulation Index	−0.090	0.493	−0.581 **	0.000	0.475 *	0.011
Motricity Index	−0.288	0.061	−0.458 *	0.019	0.128	0.624
Fugl-Meyer Assessment	−0.460 **	0.003	−0.494 *	0.014	−0.267	0.318

* *p* value < 0.05; ** *p* value < 0.005.

## Data Availability

The data supporting the findings of this study are available from the corresponding author upon reasonable request.
